# Prevention of Abuse in Animal Experimentation

**Published:** 1909-01

**Authors:** Frank Van Fleet, Joseph D. Bryant, John G. Curtis

**Affiliations:** Chairman of the Committee on Legislation; Chairman of the Committee on Experimental Medicine; Secretary of the Committee on Experimental Medicine


### CORRESPONDENCE.

**Prevention of abuse in animal experimentation.—Chairman Van Fleet of the committee on legislation and Chairman Bryant and Secretary Curtis of the Committee on experimental medicine, of the Medical Society of the State of New York, unite in an appeal on behalf of their Committees, to physicians throughout the state to oppose legislation restricting proper animal experimentation and particularly the bill proposed by the socalled society for the prevention of abuse in animal experimentation.**

*Editor Buffalo Medical Journal:*

SIR.—Will you kindly insert the enclosed in the next issue of your JOURNAL, and oblige?

Yours very truly,

(Signed) FRANK VAN FLEET,
JOSEPH D. BRYANT,
JOHN G. CURTIS.

MEDICAL SOCIETY OF THE STATE OF NEW YORK.

17 West 43d Street, New York City,
December 2, 1903.

*To the Practitioners of Medicine of the State of New York:*

The society for the Prevention of Abuse in Animal Experimentation last year caused to be introduced into the legislature of this state a bill, seriously restricting experiments on living animals. The above named society now enters the field again and proposes another bill of the same general character as that of last year. The Medical Society of the State, through its committees on legislation and experimental medicine, successfully opposed the passage of last year's measure. Acting under the resolution referring to animal experimentation, which was passed at the last annual meeting of the State Society, its committees on legislation and experimental medicine will oppose the passage of the proposed bill. We therefore urge all members of the medical profession to disapprove the measure and on its introduction into the Legislature, to work in all legitimate ways for its defeat. Specific objections to it will be presented later.

(Signed) FRANK VAN FLEET,
Chairman of the Committee on Legislation.
JOSEPH D. BRYANT,
Chairman of the Committee on Experimental Medicine.
JOHN G. CURTIS,
Secretary of the Committee on Experimental Medicine.

